# Molecular Identification and RNA-Based Management of Fungal Plant Pathogens: From PCR to CRISPR/Cas9

**DOI:** 10.3390/ijms27021073

**Published:** 2026-01-21

**Authors:** Rizwan Ali Ansari, Younes Rezaee Danesh, Ivana Castello, Alessandro Vitale

**Affiliations:** 1 Department of Ecology and Life Safety, Faculty of Geography and Ecology, Samarkand State University Named After Sharof Rashidov, Samarkand 140104, Uzbekistan; rizwans.ansari@gmail.com; 2 Department of Plant Protection, Faculty of Agriculture, Van Yuzuncu Yil University, Van 65090, Türkiye; 3 Department of Agriculture, Food and Environment, University of Catania, Via S. Sofia 100, 95123 Catania, Italy; castelloivana75@gmail.com

**Keywords:** RNAi, diagnosis, plant health, disease management, biosafety

## Abstract

Fungal diseases continue to limit global crop production and drive major economic losses. Conventional diagnostic and control approaches depend on time-consuming culture-based methods and broad-spectrum chemicals, which offer limited precision. Advances in molecular identification have changed this landscape. PCR, qPCR, LAMP, sequencing and portable platforms enable rapid and species-level detection directly from plant tissue. These tools feed into RNA-based control strategies, where knowledge of pathogen genomes and sRNA exchange enables targeted suppression of essential fungal genes. Host-induced and spray-induced gene silencing provide selective control without the long-term environmental costs associated with chemical use. CRISPR/Cas9 based tools now refine both diagnostics and resistance development, and bioinformatics improves target gene selection. Rising integration of artificial intelligence indicates a future in which disease detection, prediction and management connect in near real time. The major challenge lies in limited field validation and the narrow range of fungal species with complete molecular datasets, yet coordinated multi-site trials and expansion of annotated genomic resources can enable wider implementation. The combined use of molecular diagnostics and RNA-based strategies marks a shift from disease reaction to disease prevention and moves crop protection towards a precise, sustainable and responsive management system. This review synthesizes the information related to current molecular identification tools and RNA-based management strategies, and evaluates how their integration supports precise and sustainable approaches for fungal disease control under diverse environmental settings.

## 1. Introduction

Fungal pathogens cause most plant diseases and account for higher losses than viruses, bacteria or other microorganisms [1,2,3], and threaten around 168 crops deemed essential for human nutrition [4]. Despite extensive fungicide use and the breeding of resistant varieties, global losses to fungal pathogens remain high (10–23%) in the field and a further (10–20%) after harvest [5,6]. Major food crops such as rice, wheat, maize, soybean and potato each suffer from serious fungal or fungal-like diseases, including rice blast, stem rust, corn smut, soybean rust and late blight [7]. The yield losses attributed to these diseases represent sufficient food to sustain between 600 million and 4 billion people on a 2000 calorie diet for an entire year [8,9,10], and are expected to rise further under current climate change scenarios. Among fungi, *Fusarium* spp. rank as the most significant, together with *Magnaporthe oryzae*, *Botrytis cinerea*, *Zymoseptoria tritici*, *Colletotrichum*, *Melampsora lini* and *Rhizoctonia solani* [11,12]. Other common groups such as *Aspergillus*, *Penicillium*, *Cladosporium* and *Alternaria* also contribute to crop health decline [13]. Oomycetes are fungus-like eukaryotes within the Stramenopiles, distinct from true fungi. They have cellulose-based cell walls, predominantly diploid vegetative stages, coenocytic hyphae, and motile zoospores. For example, oomycetes such as *Phytophthora*, *Pythium*, *Peronospora*, *Plasmopara* and *Albugo* infect a wide range of hosts, with *P. infestans*, *P. sojae*, *P. capsici*, *P. ramorum* and *P. cinnamomi* among the most damaging species [14,15,16].

**Figure 1:**
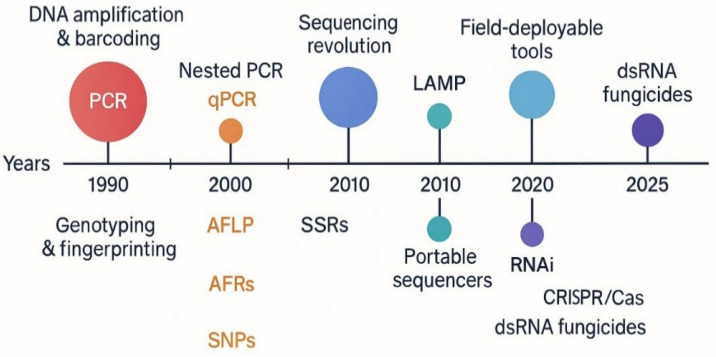
Progression of molecular tool development over time.

For effective management of these agriculturally important fungal pathogens, the first important step is accurate diagnosis and identification of plant pathogens, including fungi that severely damages plant health. Traditional diagnosis relies on morphological traits and culture-based techniques; however, these methods are slow, labor-intensive and often lack accuracy [1]. Biotrophic fungi cannot always be cultured, and results depend on individual expertise [17]. Latent infections frequently go undetected, especially when pathogen populations remain below visible thresholds [18]. Visual inspection does not give reliable early warning, and large-scale surveillance is difficult to carry out in time to prevent losses [19]. Poor sensitivity leads to delayed action or misdiagnosis [7]. In management, early gaps in knowledge often prompt broad chemical use, which encourages resistance and leaves harmful residues [2]. Rising fungicide resistance has summoned the need for accurate identification and upstream monitoring [19]. Conventional approaches still provide reference points but fall short of the precision needed in intensive production systems [20]. These limitations have driven the adoption of molecular diagnostics over the past three decades (Figure 1). DNA-based methods allow faster and more accurate detection than microscopy and culture [1]. Advances in nucleic acid extraction, internal controls, automation and field-adapted formats have helped researchers to expand the usage of molecular approaches for the correct identification and management of plant diseases [21]. Current techniques that are rigorously being used include PCR, qPCR, LAMP, FISH and microarray assays [22,23,24]. Metagenomics and next-generation sequencing provide broader community profiles and help in identifying emerging threats and monitoring probable shifts during the course of infection [25]. Portable platforms such as LAMP, lateral flow assays and recombinase polymerase amplification (RPA) offer faster field-based diagnosis and decision support [7]. RPA, an isothermal nucleic acid amplification technique wherein recombinase–primer complexes invade dsDNA and, assisted by single-strand binding proteins and strand-displacing polymerase, amplify targets at 37 °C without thermocycling, enables rapid detection of *Diaporthe* spp. Its minimal instrumentation and sample preparation render RPA ideal for point-of-care and field diagnostics of pathogens, delivering high sensitivity and specificity, with faster turnaround and minimum equipment demands [26].

Moreover, molecular methods have now been extended into management approaches. RNA interference (RNAi) blocks pathogen gene expression by degrading specific mRNAs, and thus this technique has been used in the management of several important crops effectively [27,28]. SIGS applies dsRNA externally without altering host genomes [28]. Although, substantial progress has been made in the molecular-based identification and management of fungal plant pathogens, most existing reviews tend to concentrate on either diagnostic techniques or molecular control strategies in isolation. Henceforth, we provide a comprehensive synthesis that integrates both the domains, from PCR-based fungal plant pathogen detection to RNA-guided approaches for the management of agriculturally important fungal diseases. This review addresses this gap by offering a perspective of molecular-based diagnostics and RNA-driven management, furnishing a framework to inform both future research and practical applications in enhanced crop well-being. With this impetus, the objectives of the present review are to provide a comprehensive overview of molecular diagnostic tactics for the detection and diagnosis of fungal plant pathogens and to examine different RNA-based fungal disease management strategies, offering a cohesive background for their future research and practical application.

## 2. Molecular Identification

**Figure 2:**
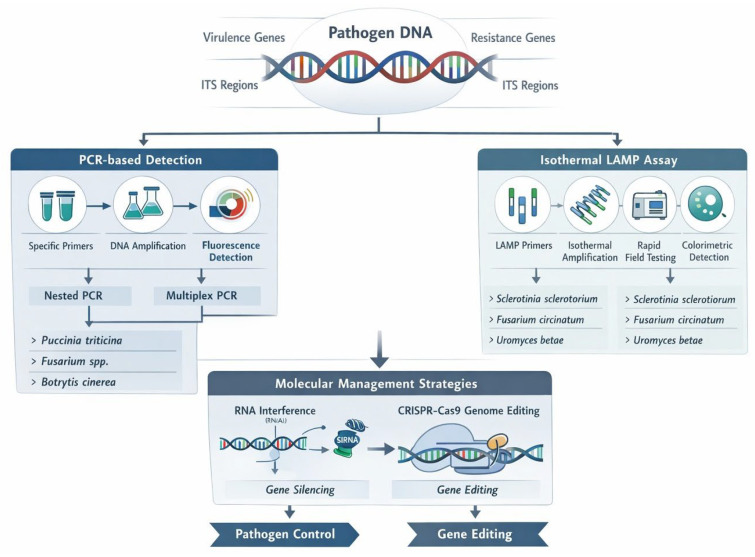
Schematic representation of the molecular diagnostic workflow for fungal plant pathogens and their targeted management using RNAi-based strategies.

Molecular-based identification of plant pathogens not only confirms their identity but also reveals key genes linked to virulence, host interaction, or fungicide resistance (Figure 2). This information guides molecular management strategies, where techniques such as RNAi or CRISPR/Cas9 are designed to silence or edit those specific genes. In this way, diagnostics provide the target, and molecular management delivers the intervention [29,30].

### 2.1. Polymerase Chain Reaction (PCR)

PCR remains a key method for detecting important fungal plant pathogens using species-specific or universal primers that enable accurate amplification and sequencing [31]. Advances following the adoption of thermostable polymerase have allowed precise identification of species and pathotypes through selective primer design, though post-amplification steps and close taxonomic similarity can limit efficiency (Table 1) [32]. Applications include assays for *Puccinia triticina* in wheat, *Phacidiopycnis washingtonensis* in apple, *Diaporthe phaseolorum* in soybean, and multiple *Fusarium* spp. in cereals [33,34,35,36]. Nested PCR enhances sensitivity by two-step amplification, while multiplex PCR detects several pathogens in one reaction, reducing time and cost [2,37]. Real-time and multiplex qPCR systems further enhance quantification and field applicability, enabling detection of pathogens like *Sclerotinia sclerotiorum*, *Colletotrichum truncatum*, and *Corynespora cassiicola* at very low incidence levels [38]. The nuclear ribosomal operon remains the main target for fungal diagnosis, with ITS, 28S (nrLSU) and 18S (nrSSU) regions used for amplification and identification [39]. The ITS region offers high variability for distinguishing isolates and can be paired with protein-coding genes for more accuracy. However, PCR reliability is affected by DNA extraction efficiency, sample inhibitors and reaction conditions [40,41]. qPCR enables real-time detection and quantification of fungal DNA or RNA through fluorescence-based monitoring during amplification [42]. Using sequence-specific primers, it measures target abundance via cycle threshold values and provides greater sensitivity, speed and reproducibility than conventional PCR [43,44]. Detection limits can reach as low as 2 fg of genomic DNA, equivalent to a single spore [45,46].

### 2.2. Loop-Mediated Isothermal Amplification

Though sequencing methods give unrivalled resolution, their expense and complexity prevent them from being used routinely in diagnostic work, which can be served by simpler isothermal assays in field situations. LAMP is one of the useful methods for the detection of fungal pathogens, especially where laboratory facilities are lacking. It operates at constant temperatures of 60–65 °C, amplifying the amount of DNA available 10^10^ times in an hour at most, using six primers directed against specific genomic regions, and can be run in a water bath [34,45]. The method has the advantage of being free of a thermocycler and is, therefore, suited to rapid low-cost field diagnostics based on simplified methodologies, with reactions being completed in less than half an hour [1,18,55]. The method offers analytical sensitivity a thousand times better than existing measures and gives a measure of the intrinsic limitations of existing diagnostics. Its analytical sensitivity equals that of qPCR and surpasses conventional PCR, detecting as little as 10–100 fg of DNA, particularly in heat-dry RT-LAMP formats [34,56]. Visual detection through color change, using hydroxyl naphthol blue or calcein, further supports on-site diagnosis [57,58]. Overall, the incorporation of portable LAMP systems, along with lateral flow and recombinase polymerase amplification, has driven molecular diagnostics towards accurate pathogen detection under different field conditions [7,59].

## 3. Marker and Fingerprinting Approaches

Molecular markers are nucleotide sequences that enable us to detect different plant pathogens (Table 2). Molecular markers like RAPD, RFLP, AFLP, SSR, and SNP are applied to diagnose and identify fungal plant pathogens and assess intraspecific variation, population structure, and genetic diversity [60,61,62]. The marker selection depends on the organism, objectivity, and desired resolution [63]. For the last two decades, these PCR- and restriction-based techniques have become essential to fungal population studies that support taxonomic identification and evaluation of genetic differentiation among different species.

### 3.1. Random Amplified Polymorphic DNA (RAPD)

RAPD employs short unique arbitrary primers (8–12 bases) that bind to different genomic loci that generate species-specific amplification profiles [70]. It is less costly, requires comparatively little DNA, and is used to study genetic diversity in some commonly occurring fungi such as *Fusarium*, *Elsinoë*, and *Venturia* [71,72,73]. Despite its simplicity, RAPD exhibits low reproducibility and limited resolution [70,74]. The conversion of RAPD bands to SCAR markers increases specificity and consistency of reagents; for example, assays for *Pseudofabraea citricarpa* would be able to detect 0.1 ng of DNA [75,76]. RAPD has supported population studies of *Alternaria*, *Ophiostoma*, *Leptosphaeria maculans*, and *Erysiphe pisi*, revealing variable patterns of diversity [77,78,79].

### 3.2. Restriction Fragment Length Polymorphism (RFLP)

RFLP determines sequence variation by using restriction enzymes to produce different fragment patterns that distinguish among different strains of fungi [80]. It is one of the first molecular instruments used in population analysis, especially on pathogens like *Ophiostoma ulmi* and *Ceratocystis* spp. [78]. In spite of the fact that the technique has rather high discriminatory power, it has been shown in studies of *Fusarium oxysporum* that it requires large amounts of pure DNA and is relatively less efficient in comparison with PCR-based or sequencing systems [81]. The method has been applied in population typing of pathogenic fungi, such as in the use of RG57 probes to detect the presence of pathogenic organisms, and in ITS-RFLP-PCR assays for detection of the pathogenic species *Rhynchosporium* [82].

### 3.3. Amplified Fragment Length Polymorphism (AFLP)

AFLP analyzes genomic variation by digesting DNA with restriction enzymes, ligating adaptors to fragment ends and selectively amplifying subsets using adaptor-specific primers [71]. It combines restriction and PCR principles, detects 50–100 polymorphic fragments per assay, and provides high reproducibility without prior sequence data [63,83]. Although this method needs intact DNA and involves several highly technical steps, it remains pivotal for the characterization of fungal population structure and differentiation of species such as *Fusarium commune* from *F. oxysporum* [84]. Determination of AFLP fragments for SCAR markers has further enhanced its diagnostic ability, as seen in *Puccinia striiformis* strain typing [62].

### 3.4. Simple Sequence Repeats (SSRs)

SSRs or microsatellite markers consist of short tandem repeats distributed in nuclear and organellar genomes. The codominant inheritance, high polymorphism and reproducibility render SSRs valuable for mapping, population characterization and accurate pathogen identification [60,85]. Though they are costly and time-consuming to develop, SSRs offer greater resolution than RAPD or AFLP, and detect considerable genetic variation within populations [86,87]. This technique has enabled high-resolution genotyping of *Phytophthora infestans*, pathotype differentiation in *Puccinia graminis* f. sp. *tritici*, and diversity analyses in *Verticillium dahliae* and *Pseudoperonospora cubensis* [88,89,90,91].

### 3.5. Single Nucleotide Polymorphisms (SNPs)

SNP markers actually target single base variations in the genome, offering high reliability, stability and abundance for characterizing population structure and diversity in fungi that cause diseases in plants [92,93]. Advances in sequencing technologies such as WGS, RAD-seq and genotyping-by-sequencing have enabled efficient SNP discovery; however, these tools are costly and limited to regions near restriction sites [94,95]. SNP markers show strong discriminatory capacity (*Erysiphe* and *Ustilaginoidea virens*), where many variants distinguished closely related taxa and defined multiple multilocus types [86,93]. In *Phytophthora capsici*, genome-wide SNP analyses identified loci associated with mating type and seasonal variation, reinforcing their diagnostic and population-level value [96,97].

## 4. Sequencing and Barcoding

DNA barcoding uses short genetic regions, usually of 500–800 base pairs, to identify fungal species through sequence comparison with verified references [98,99]. This approach has redefined fungal taxonomy by exposing cryptic diversity and separating species once grouped by morphology. The ITS region serves as the primary barcode, while translational elongation factor 1α is a key supplementary locus, though multilocus analysis is often needed for accurate species resolution [98,100].

### 4.1. ITS as the Primary Fungal Barcode

The ITS region of nuclear rDNA serves as the primary fungal barcode because its conserved and variable segments enable species-level distinction across taxa [1,101,102]. ITS sequencing has been used for identifying over 80% of fungi infecting bast fiber crops and for confirming pathogens such as *Colletotrichum gloeosporioides*, *C. higginsianum* and *C. phormii* [1]. The ITS2 subregion has proved valuable for the detection of *Fusarium* spp. that cause diseases on cereals [102].

### 4.2. Translation Elongation Factor 1α (TEF-1α)

The TEF-1α gene serves as the main secondary barcode for fungi, complementing ITS data to improve species-level resolution [103,104]. It has proved particularly effective in *Fusarium*, where morphology offers limited discrimination, providing accurate identification and reliable metabarcoding results across field isolates [105,106,107]. TEF-1α also assists in distinguishing other crop pathogens, and its use alongside ITS, applied when ITS similarity falls below 98.5%, has become standard for precise fungal diagnosis [103,108].

### 4.3. Multi-Locus Sequence Typing (MLST)

MLST sequences several housekeeping genes, improves resolution in fungal identification beyond single-locus methods, and is now a standard for phylogenetic and epidemiological studies [90,109,110]. By using loci such as ITS, ACT, TUB2, CAL, and GAPDH, MLST has clarified species boundaries in *Colletotrichum* and *Cercospora*, where morphology and individual markers fail [1,111]. It also supports population analyses through the detection of clonal and recombination events, as found in *Tilletia indica* through multilocus data from seven genes [112].

## 5. High-Throughput Sequencing (HTS)

HTS, also known as next-generation sequencing (NGS), has revolutionized pathogen detection and diagnosis with higher speed [45,102]. Unlike conventional diagnostics tools focused on individual isolates, HTS provides metagenomic insights into entire microbial communities, capturing a wide range of taxa and facilitating the identification of obligate or previously unrecorded fungi [113,114]. The method permits genome sequencing without sequence data availability, which is essential for identification of new or emerging pathogens [115]. HTS encompasses several platforms, like quantitative and droplet digital PCR for designed probes and nanopore-based systems that offer affordable, real-time sequencing [116,117]. Long-read approaches, including nanopore sequencing and SMRT, yield full-length ribosomal regions and decrease assembly-related ambiguities; however, cost and error rates still constrain widespread application [102,118]. The continuous expansion of public fungal genome databases enhances diagnostic precision and enables population-level studies that identify nucleotide polymorphisms and structural variants linked with virulence and evolution [45,101,119].

### 5.1. Amplicon-Based Metabarcoding

Amplicon-based metabarcoding targets conserved genomic regions to identify and profile fungal communities [99]. The ITS region, which includes ITS1, 5.8S, and ITS2 between the 18S and 28S rRNA genes, serves as the main barcode for species-level classification, while loci such as beta-tubulin, TEF-1α, and RNA polymerase II subunit (RPB2) improve taxonomic resolution in certain plant-pathogenic groups [70,120]. Metabarcoding has proven effective for detecting soilborne fungi such as *Rhizoctonia*, *Fusarium*, *Verticillium*, *Sclerotinia*, *Pythium*, and *Phytophthora*, without requiring host tissue or prior pathogen assumptions [121]. It has been applied in diverse environments, including rice agroecosystems to monitor seasonal shifts of *Pyricularia*, *Bipolaris*, *Cladosporium*, *Alternaria*, and *Myrothecium*, as well as in irrigation water and forest surveys where numerous *Phytophthora* species have been detected [122,123,124]. Moreover, ITS1 and ITS2 analyses identified broader fungal diversity, with ITS2 exhibiting superior resolution for *Fusarium* species, while the same approaches have been used for the identification of coffee rust lesions [102,121]. Long-read sequencing systems facilitate recovery of the complete ITS region in sole reads, reducing assembly bias, but require higher costs, DNA quality, and computational effort, while short-read methods are preferred for biodiversity studies. Consequently, long-read platforms hold promise for diagnostic applications that require fine taxonomic accuracy [125].

### 5.2. Shotgun Metagenomics and WGS for Virulence Gene Discovery

Shotgun metagenomics sequences the whole DNA content of ecological samples without PCR amplification, which enables detection of both culturable and unculturable fungi and the reconstruction of complete or near-complete genomes of microorganisms [121,122]. WGS improves this process by allowing strain-level identification, for example, in *Calonectria pseudonaviculata*, where Illumina MiSeq data exhibited a 51.4 Mb genome containing diagnostic SNPs [45]. Large-level genomic analyses explore genetic variation like insertions, deletions, and structural changes associated with virulence and evolution [119]. Transcriptomic studies further enhance diagnostic ability: RNA-Seq of *Puccinia striiformis* f. sp. *tritici* in wheat clarified the diversity of yellow rust populations, while metatranscriptomic profiling of Verticillium wilt in olives demonstrated a polymicrobial complex rather than a single causal agent [126,127].

### 5.3. Portable Sequencers (MinION)

The MinION sequencing platform provides rapid detection of plant pathogens through both amplicon and metagenomic approaches, completing workflows from DNA extraction to analysis in about 2.5 h [7,128]. It has identified *Candidatus Liberibacter asiaticus* and plum pox virus directly from tissues often within the first sequencing reads [129,130]. In fungal diagnostics, *Calonectria pseudonaviculata* infections produced over 9% target reads, allowing strain-level identification, while full-length ITS regions were sequenced in single reads, avoiding short-read fragmentation [120,131]. The MARPLE system, which targets variable genes rather than whole genomes, has reduced data volume and enabled rapid detection of *Puccinia striiformis* f. sp. *tritici* strains within 48 h in East Africa [7,132]. Portable sequencers have also revealed causal agents of wheat stripe rust (*Puccinia striiformis* f. sp. *tritici*), Septoria tritici blotch (*Zymoseptoria tritici*), and yellow leaf spot (*Pyrenophora tritici repentis*) [133]. Nanopore sequencing continues to advance as a practical tool for early disease detection and surveillance [116,117].

### 5.4. Artificial Intelligence and Machine Learning Integration

The incorporation of artificial intelligence (AI) and machine learning (ML) in fungal pathogen diagnostics improves overall accuracy, speed and predictive capability [134]. Platforms using AI include advanced imaging, biomarker analysis and point-of-care testing, offering considerable improvements over conventional techniques [135]. MALDI-TOF mass spectrometry benefits from neural network integration that further improves data interpretation. Convolutional neural networks identify isolates of *Aspergillus flavus* with over 93% accuracy without further molecular assays [136]. Applications of computer vision and deep learning in histopathological studies differentiate *Aspergillus* from Mucorales species with high efficacy [103,137,138]. Spectroscopic methods integrated with ML assist in rapid fungi identification. Raman spectroscopy integrated with deep learning autoencoders achieves 97% accuracy in bacterial models with one-second acquisition; however, fungal applications remain limited [139,140,141]. Surface-enhanced Raman spectroscopy linked with backpropagation neural networks achieves 98.23% identification accuracy, offering cost-effective and rapid fungal species detection [142]. LC-MS/MS with AI algorithms empowers fast species prediction, biomarker identification and quantification of plant pathogens [143]. Certain levels of fungal species coverage and field validation create problems in practical implementation; however, expanding annotated spectral and imaging datasets and integrating multi-platform AI pipelines could qualify broader, field-ready diagnostic applications. Briefly, AI and ML show strong potential for fungal plant disease diagnosis, but progress remains constrained due to limited, well-annotated, and field-diverse datasets for many pathogens. Models often fail to generalize across cultivars, environments, and disease stages, and thus their low interpretability restricts confidence among researchers. Future work should focus on user-friendly models, good data curation, and robust validation under field conditions.

## 6. Bioinformatics

Few databases specialize in the identification of plant pathogenic fungi; however, general fungal databases contain extensive taxonomic and sequence data [144]. Resources such as onestopshopfungi.org and the GOPHY platform (plantpathogen.org) dispense curated molecular and morphological information including updated taxonomy and DNA barcodes for plant pathogens [145,146]. However, their continuous maintenance remains a critical challenge due to frequent taxonomic revisions and ongoing refinement of genomes [147,148]. Bioinformatics repositories constitute an essential infrastructural backbone for contemporary molecular applications in mycological and phytopathological research. Widely used resources, including GenBank hosted by the National Center for Biotechnology Information, the Nucleotide Sequence Database Collaboration maintained by the European Bioinformatics Institute, and MycoBank, provide authoritative platforms for the registration of fungal taxonomic novelties and the archival of nucleotide sequence data derived from plant-pathogenic fungi, thereby strengthening molecular-based diagnostic frameworks and improving overall species identification accuracy [45]. In culture-based genomic investigations, sequence datasets generated through conventional molecular protocols are subsequently curated and analyzed using user-oriented bioinformatics software such as MEGA 12.1 and BioEdit 7.2, which enable sequence trimming, multiple alignment, and comparative analyses against publicly available databases to support reliable species-level identification [149].

Notwithstanding the considerable advancements introduced by molecular and bioinformatics-driven methodologies, their routine implementation in fungal pathogen diagnostics remains hindered by a number of inherent technical and methodological constraints. In particular, DNA-based identification strategies are frequently limited by reduced analytical sensitivity when assays are designed around single-copy nuclear loci, as well as by their restricted capacity to resolve cryptic taxa that remain indistinguishable under commonly employed diagnostic marker systems [11]. Collectively, these limitations somehow compromise the robustness and accuracy of pathogen detection, especially in species complexes where high genetic similarity undermines discrimination using traditional molecular targets [11].

## 7. Reference Base and Data Curation

Reference databases for fungal pathogens serve importantly in pathogen diagnoses, research and management. The Pathogen–Host Interactions (PHI) database solely remains the principal platform, documenting over 3000 experimentally validated genes that are linked to pathogenicity and virulence from 160 species of 103 plant pathogens [150]. The DFVF complements it with 2058 genes from 228 strains across 85 genera, out of which 539 factors are plant-associated [151]. The database repositories of genomes, like MycoCosm, provide full genomes, secretome data, and tools for cross-species analysis [152]. Additionally, manual database curation ensures data reliability but limits scalability, prompting community-based approaches like the VBI Microbial Database and collaborative annotation projects for *Phytophthora* and *Blumeria graminis* [153,154]. Misannotations and taxonomic errors are common due to genome complexity and frequent reclassification, which needs automated quality checks [155,156]. Emerging tools like T-BAS give dynamic phylogenetic frameworks for *Phytophthora* identification, while MARDy curates antifungal resistance markers, but still needs broader automation and expansion [157].

The analysis of fungal genomes now depends on integrated multi-stage pipelines that integrate assembly, annotation, and comparative mapping to produce and refine reference genomes [158,159]. Specialized workflows such as DADA2 and TheiaEuk regulate amplicon and WGS, respectively, which supports data filtering, classification, and species-level identification without extensive analyses and programming [160,161,162]. Although platforms like Pacific BioSciences Sequel yield greater fidelity than MinION, inconsistency in data quality remains [128]. Comparative genomics platforms like EuPathDB increase analytical reproducibility through Galaxy-based workflows, though curated standards information for fungal datasets is to be observed clearly [163]. Recent annotation pipelines incorporate virulence and metabolic gene screening against PHI-base; however, the limited coverage of tools like g:Profiler, DAVID, and WebGestalt restricts interpretation for non-model fungi [164,165,166].

## 8. How Molecular Markers Reshaped Taxonomy of Cryptic Species

Modern approaches including DNA sequencing redefined fungal taxonomy by determining ambiguities that morphological traits could not address. This has allowed classification to reflect evolutionary relationships through molecular markers like the ITS region [167,168]. Molecular phylogenies revealed hidden diversity in common fungi such as *Alternaria*, *Botryosphaeria*, *Cercospora*, *Diaporthe*, and *Fusarium*, where morphologically identical isolates were found to be genetically dissimilar [11,169,170]. Accurate identification now depends on multi-gene datasets incorporating *rpb1*, *rpb2*, *tef1*, *β-tubulin*, and *calmodulin*, as ITS alone rarely distinguishes closely related taxa [11,171,172,173,174]. In *Fusarium*, tef1-α is now considered the most important frontline marker for species identification due to differences in its sequences between closely related species that resolve species boundaries, while ITS often cannot [175]. Molecular databases have prompted revisions of major taxa. For example, *Mycosphaerella* has been divided into families like *Teratosphaeriaceae*. Also, molecular databases have fixed the ambiguities of polyphyletic genera, including *Fusarium*, *Colletotrichum* and *Curvularia* [168,176,177,178]. The Genealogical Concordance Phylogenetic Species concept explained an outline for identification of cryptic taxa [11]. Phylogenetic analysis reconsidered *Colletotrichum* spp. complexes, describing a wide range of isolates within a species [179]. NGS registered significant improvements in strain resolution in the *Fusarium graminearum* complex and exposed cryptic diversity within *Macrophomina phaseolina* and *Lasiodiplodia* [11,180,181,182]. Studies on *Rhizoctonia solani* reported that AGs represent distinct evolutionary units, enabling host-specific management approaches [183]. Moreover, in the *Ceratocystis fimbriata* complex, MLST approaches using ITS and TEF1-α loci unraveled 23 sequence and 22 allele types, showing genetic diversity in three different clades [184].

## 9. Molecular Approaches for Disease Management

### 9.1. RNA Interference

RNAi provides a precise means of fungal disease management by using 20–24 nucleotide RNA molecules to silence essential genes through targeted mRNA degradation (Table 3). dsRNA matching key fungal sequences suppresses vital processes such as cell wall formation, metabolism and virulence, while avoiding harm to the host or beneficial microbes. Its success depends on appropriate gene selection, stable delivery and persistence within the plant–pathogen system [185].

#### 9.1.1. Mechanisms

RNAi in fungi depends on Dicer-like and Argonaute proteins that process dsRNA into 21–25 nucleotide siRNAs and direct their degradation of complementary mRNAs, blocking protein synthesis and impairing key cellular functions [191,192]. Small RNAs move between host and pathogen through extracellular vesicles: plants transfer silencing RNAs to suppress fungal virulence genes, while pathogens such as *Sclerotinia sclerotiorum* and *Botrytis cinerea* deliver sRNAs to inhibit host defenses [28,193]. Two control methods exploit this mechanism: Host-Induced Gene Silencing (HIGS), where transgenic plants produce pathogen-targeted hairpin RNAs, and Spray-Induced Gene Silencing (SIGS), which applies dsRNA externally and avoids genetic modification [194,195]. Nanocarrier-based delivery systems, including liposomes and dendrimers, now enhance dsRNA stability and uptake in fungal cells [196,197].

#### 9.1.2. Target Genes and Pathways

RNAi-based control of fungal pathogens depends on selecting essential targets involved in viability, virulence, or metabolism (Table 4). Targeting RNAi machinery genes, including *ARGONAUTE* and *DICER*, through SIGS has also suppressed infection in cereals [189,198]. Species-specific targets, such as *FolRDR1* in *Fusarium oxysporum* f. sp. *lycopersici*, resulted in considerable alleviation of fusarium wilt and also limited off-target effects. HIGS in banana, maize, and tobacco reduced disease severity and mycotoxin levels through silencing of transcription factors or chitin synthase genes [199,200]. Broader SIGS application suppressed *Botrytis cinerea*, *Sclerotinia sclerotiorum*, and *Rhizoctonia solani*, though *Colletotrichum gloeosporioides* and *Trichoderma virens* showed no and limited uptake, respectively [188]. Recent work has extended RNAi to powdery mildews and viruses, where silencing of lipid metabolism and abscisic acid pathway genes restricted fungal colonization, and dsRNA application against *Zucchini yellow mosaic virus* protected the plants from virus infection [201,202].

### 9.2. Host-Induced Gene Silencing

HIGS employs plant RNAi pathways to silence essential fungal genes by expressing matching dsRNA in the host [211]. Upon infection, the pathogen absorbs these molecules, activating its own RNAi machinery to suppress target gene expression, thereby limiting growth and virulence [212]. Effective resistance depends on selecting pathogenicity or survival genes that minimize non-target effects, offering precise, chemical-free control across multiple pathogens sharing conserved pathways.

#### Mechanisms

HIGS functions through RNA transfer between plants and fungal pathogens, though the precise transport routes remain uncertain. Evidence indicates that RNA moves as free molecules, through carrier proteins, or via extracellular vesicles that deliver sRNAs from host to pathogen, where they activate fungal RNAi machinery [213]. This cross-kingdom exchange enables plants to silence genes essential for fungal virulence. Successful applications include CYP3RNA-mediated silencing of *CYP51* genes (*FgCYP51A*, *FgCYP51B*, *FgCYP51C*) in *Fusarium graminearum*, which disrupts ergosterol synthesis and limits infection, and *Chs3b* silencing that enhances resistance to *Fusarium*-induced diseases. Similar suppression of *Avra10* in *Blumeria graminis* improved resistance in barley and wheat [214,215,216].

### 9.3. Topical dsRNA

Topical application of dsRNA has emerged as a molecular strategy for controlling fungal plant diseases by harnessing endogenous RNAi pathways in the pathogen. When dsRNA is placed on plant tissue or directly contacts fungal structures, the pathogen can internalize these molecules and process them through its own Dicer enzymes. The resulting siRNAs then direct the degradation of complementary messenger RNA, leading to targeted suppression of essential fungal genes [217,218].

#### Mechanisms

After entering a fungal cell, dsRNA is processed by Dicer-like endonucleases into siRNAs that guide gene silencing through Argonaute-containing complexes [219,220]. These siRNAs can arise within the pathogen or transfer from plant cells already processed by host Dicer enzymes, allowing degradation of fungal transcripts essential for infection and survival [196,219,221]. Topical dsRNA may also reduce fungal growth through stress responses or by activating plant pattern-triggered immunity that produces reactive oxygen species. RNA-binding proteins such as AGO, DCL, ARGONAUTE2 and GRP7 maintain siRNA stability and movement across the plant–fungus interface [197,222].

### 9.4. Pitfalls of dsRNA Applications Under Field Conditions

dsRNA offers a modern molecular approach for controlling fungal plant pathogens through RNAi. After application to the pathogen, the dsRNA is processed into siRNA molecules that silence genes required for growth, reproduction or pathogenicity [223]. This strategy provides high target specificity, avoids chemical residues, and has the potential to manage fungicide-resistant strains [224]. One major barrier to SIGS is the rapid loss of dsRNA integrity under field conditions. Ultraviolet light, temperature fluctuations, pH changes and microbial nuclease activity cause degradation within short periods after application [204,205]. Biological activity may decline within 1 h under UV exposure [98]. Temperature affects the stability of dsRNA, which is usually stable between 25 and 37 °C. Thus, raised temperatures degrade the dsRNA, with more than 45% loss observed after 6 h at 50 °C [225]. Moreover, degradation in soil also occurs and is even faster. Studies report half-lives of 15–28 h with non-significant effects of soil texture or pH [226,227]. Biological activity becomes undetectable nearly 2 days after application, as microbial nucleases trigger degradation [228,229]. Microorganisms harbored in the phyllosphere and rhizosphere drive this breakdown process efficiently [230]. In addition, the economics of dsRNA production have improved markedly and given hope to researchers. In 2008, costs approaching USD 10,000 per gram restricted large-scale commercial use [231]. However, recent advances in synthesis methods have further decreased this figure to about USD 100 per gram in vitro (less than USD 1 per gram in cell-free systems) [232]. With realistic field application rates near 10 g per hectare, the cost of dsRNA is now very close to that of conventional chemicals [233]. However, scaling up to large-scale synthesis is a further challenge that needs to be investigated without leaving any details untouched. In vitro transcription results in only microgram quantities and is found to be expensive [234,235,236]. Thus, many studies still rely on commercial kits that do not meet field-scale requirements. Moreover, field application of dsRNA raises further practical constraints. For example, the plant cuticle, cell wall and plasma membrane behave as physical barriers that limit foliar entry of dsRNA [188,237]. Factors like surface waxes, stomatal density and leaf wettability also influence uptake of dsRNA [28,238,239]. RNA uptake ability varies greatly among plant pathogens. Efficient uptake occurs in *Botrytis cinerea*, *Sclerotinia sclerotiorum*, *Rhizoctonia solani*, *Aspergillus niger* and *Verticillium dahliae*. In contrast, no uptake occurs in *Colletotrichum gloeosporioides*, and uptake remains weak in beneficial fungi such as *Trichoderma virens* [188,240]. Oomycetes such as *Phytophthora infestans* show limited uptake that varies by cell type [188,241]. The success of SIGS depends on the pathogen’s uptake efficiency [188]. Weather and environmental exposure further reduce the effectiveness of RNA application, especially under field conditions. Rainfall, heat, UV radiation and microbial activity remove dsRNA from leaf surfaces or degrade it before uptake occurs [28].

## 10. CRISPR/Cas9 and Genome Editing

CRISPR/Cas9 offers targeted genome modification in both fungal pathogens and host plants, and its application follows two main strategies. One approach focuses on altering genes in the pathogen itself, particularly those linked to virulence, effector activity or metabolic pathways required for host invasion [242]. The other strategy edits host susceptibility genes that pathogens rely on during infection, allowing resistance development without the addition of external genetic material [243].

### 10.1. Mechanisms

#### 10.1.1. Pathogen Gene Editing Mechanisms

CRISPR-based editing modifies pathogen genomes by inducing targeted DNA breaks through Cas9–RNA complexes, followed by repair that disrupts or replaces specific sequences via nonhomologous end joining or homology-directed repair [244]. Catalytically inactive Cas9 fused with transcriptional repressors can also silence genes without altering DNA, as demonstrated in *Magnaporthe oryzae* and *Ustilaginoidea virens* [244]. Gene disruptions that alter virulence, such as *PpalEPIC8* in *Phytophthora palmivora*, markedly reduce pathogenicity, while modification of avirulence loci, like *AvrLM7* in *Leptosphaeria maculans*, clarifies host recognition mechanisms [245,246].

#### 10.1.2. Host Susceptibility Gene Editing Mechanisms

CRISPR/Cas9 editing of host susceptibility genes targets plant loci that facilitate pathogen invasion, strengthening innate defenses such as pattern- and effector-triggered immunity [247,248]). The MLO gene family represents a key susceptibility group; loss-of-function mutations confer broad resistance to powdery mildew across crops. Disruption of TaMlo in wheat and deletion of a short SlMLO1 fragment in tomato produced resistant lines without off-target effects [249,250]. Moreover, CRISPR/Cas9 tools offer a very powerful approach for the management of fungal plant pathogens by the production of crop varieties containing durable resistance. For example, using the CRISPR/Cas9 technique, wheat lines that exhibit resistance to powdery mildew have been developed [251].

The CRISPR/Cas9 system exhibits certain limitations in developing disease-resistant plants. One major challenge arises from the direct targeting of susceptibility genes, which may impose a fitness cost due to their genetic linkage with other loci essential for plant growth and development [247]. Moreover, disruption of a susceptible gene can perturb its associated metabolic or signaling pathways, potentially affecting downstream products and leading to deficiencies in key micronutrients or observable phenotypic alterations. The severity of these fitness costs can vary depending on the specific susceptible gene targeted and the functional roles of associated factors, including susceptibility facilitators, defense suppressors, or pre-penetration components involved in pathogen replication. Strategies to mitigate these effects include the design and introduction of susceptible gene variants with minimal collateral effects, promoter targeting to generate precise allelic modifications, or the application of base editing approaches to achieve targeted nucleotide changes without introducing double-strand breaks [244,247]. Non-target effects remain the main challenges in CRISPR applications for enhancing resistance against a wide array of fungal diseases. These errors take place when the system edits genomic regions that resemble the intended target [252,253,254]. Such unintended edits may lead to genomic instability, cell death or unwanted traits that could pass to the next generation [255,256,257]. However, most documented off-target changes are small insertions, deletions or point mutations, while large deletions appear rare [255,256].

## 11. Molecularly Informed Breeding

Molecularly informed breeding shifts plant improvement from phenotype-based selection to decisions guided by genetic data. By using molecular markers and genome analysis, breeders can locate DNA regions associated with resistance traits and retain them during selection. This way, it is possible to track resistance level against the fungal pathogens without waiting for visible infection, allowing accurate and timely identification of lines [258].

### 11.1. Marker-Assisted Selection (MAS)

MAS utilizes DNA markers coupled to quantitative trait loci to trace and transfer resistance alleles into elite breeding lines, often through marker-assisted backcrossing that retains desirable characters while introducing target loci [259,260]. It remains highly effective for major loci such as *Fhb1*, which confers Fusarium head blight resistance in wheat, including durum [261,262].

### 11.2. Genome Selection

Genomic selection explains the scope of selection through the use of genome-wide marker data to predict resistance without depending on individual markers [260]. Genomic selection is a method that uses data from the entire genome to predict how well a plant will resist diseases, rather than relying on just one specific marker. This approach improves the accuracy of selecting plants for important traits, such as reducing harmful substances like mycotoxins, and can speed up the breeding process. Prediction models based on whole-genome markers enhance the selection accuracy for characters like mycotoxin accumulation and curtail breeding cycles [261]. This strategy enhances genetic gain for traits regulated by multiple loci of small effect and offers clear benefits over traditional selection approaches [263]. Overall, it offers considerable advantages for developing stronger, healthier crops against plant pathogens.

### 11.3. Introgression

Introgression approaches transfer resistance genes from wild relatives into cultivated crops, expanding the genetic base for durable defense. For example, *Lr34*, *Lr46* and *Sr57* from *Triticum turgidum* and *Aegilops* spp. provide broad-spectrum and long-lasting defense by modulating systemic resistance pathways in wheat against major rusts, and the incorporation of Pi54, Pi5, *Pi2* and *Pi9* for rice blast resistance has been observed in 20 traditional rice varieties [264,265,266,267]. Modern approaches integrate introgression with MAS and genomic selection to stack resistance loci without compromising plant growth, biomass and yield [265].

## 12. Molecular Insights Guiding Biocontrol

Application of omics-based techniques has improved fungal disease management potential. Genome-level analyses now aid the selection of biocontrol agents through the identification of genes linked to antagonistic activity against target plant pathogens. Transcriptomic studies offer complementary evidence by showing how gene expression in both the pathogen and the biocontrol organism modulates during their interaction, revealing pathways involved in suppression or defense [268]. Metabolomic profiling further adds a dimension by characterizing antifungal compounds and mapping the biochemical exchanges that influence the pathogen’s viability [269].

Genomic tools now help in selecting biocontrol agents by identifying genes linked to antagonism, unravelling novel gene functions, and allowing comparison across beneficial microbes [270]. Metabarcoding of soil and root microbiome under a wide array of cultivation systems links microbial diversity with agronomic practices, enabling systematic screening of isolates such as *Trichoderma* spp. against *Fusarium xylarioides* [271,272,273]. Transcriptomics and proteomics analyses further reveal functional responses, and show how the gene and protein expression change in relation to secondary metabolites and lytic enzymes that break down fungal cell walls [274]. Metabolomic profiling recognizes antifungal compounds, antibiotics, and signaling molecules, explaining mechanisms of pathogen suppression and plant defense system activation [275].

Integrated omics approaches enable molecular events to be investigated throughout regulatory layers. Studies in *Phyllosticta citricarpa* and *Bacillus subtilis* CF-3 reveal that volatile compounds and bacterial metabolites change fungal metabolism, gene expression, and membrane stability [276,277]. Also, metabolomics remains central for strain selection, distinguishing *Trichoderma* spp. with strong antagonistic potential and identifying key discriminant metabolites through LC–MS and principal component analysis [272,278]. Insights from multi-omics also disclose plant–microbe interactions. Root colonization by *Trichoderma* spp. and associated metabolites triggers and activates genetic and biochemical defense systems against diverse stresses, though systemic responses remain difficult to unravel due to temporary transcriptional changes [272,279].

The overall strategy from diagnosis to management of fungal plant pathogens has transformed from traditional detection to modern molecular tactics that combine genome analysis with targeted interference [280]. Early diagnosis depends on qPCR, LAMP, nanopore sequencing, etc., which deliver rapid and accurate identification of fungal pathogens, allowing swift disease monitoring and management [261]. The need for reliable molecular diagnostics is high because fungal diseases threaten the global food supply, causing considerable yield loss during cultivation and also after harvest [281]. After laboratory confirmation of the pathogen, genome analysis through functional genomics and multi-omics tools enables researchers to identify genes essential for growth or virulence [261,282]. This information guides the design of precise RNA constructs that silence target genes while avoiding effects on non-target organisms [282]. Research into cross-kingdom RNAi has shown that plants and pathogens exchange small RNAs, and that some fungal pathogens, including *Botrytis cinerea*, release these molecules into plant cells to suppress host defense [28]. The final stage of this system uses targeted management tools such as HIGS-, SIGS- and CRISPR/Cas9-based tactics [261,280,281]. CRISPR/Cas9 introduces durable resistance by altering host genes linked to immunity, while RNA-based methods silence pathogen genes without altering plant DNA [283]. This combination offers a promising alternative to chemical pesticides and provides a flexible strategy for controlling new pathogen variants while supporting environmental sustainability [28,282].

## 13. Conclusions

Molecular methods have shifted fungal pathogen management from reactive to predictive. PCR, sequencing and marker-driven assays enable precise and rapid identification, while RNA-based strategies target essential pathogen genes and reduce reliance on broad-spectrum chemicals. When molecular identification directs RNA-based control, detection and treatment operate as one system and allow decisions at the earliest point of pathogen activity. Future progress will rely on cross-disciplinary research to understand the essence of RNAi-based, agriculturally important plant disease management. Bioinformatics refines diagnostic markers and RNA targets. CRISPR/Cas9 technology offers portable diagnostics with near-instant decision support. AI strengthens disease prediction and facilitates real-time monitoring in the field. Limited field validation and narrow datasets continue to restrict deployment at scale, yet enlarging shared annotated datasets, together with coordinated multi-location trials, can move these methods from research to routine use. Integrating molecular diagnostics with RNA-based control strategies does not only improve disease management. It also establishes a new standard of precision agriculture where fungal pathogens are identified early, targeted accurately and controlled sustainably.

## Figures and Tables

**Table 1:** Different versions of PCR used for detection of agriculturally important fungal plant pathogens.

Different PCR	Fungi Diagnosed	Host	Geographic Locations	Objectives	References
Nested PCR	*Pilidiella granati*	Pomegranate	China	Rapid PCR-based detection of *Pilidiella granati* in pomegranate.	[47]
Nested PCR	*Phytophthora nicotianae*, *P. citrophthora*	Citrus	Italy	Nested PCR-based detection of *Phytophthora* spp. in citrus roots and soil.	[48]
Nested PCR	*Phytophthora cactorum*	Strawberry	USA	PCR-based detection of *Phytophthora cactorum* in strawberry plants.	[49]
End-point PCR	*Phacidiopycnis washingtonensis*, *Sphaeropsis pyriputrescens*	Apple	USA	Development of PCR assays for early detection of *Phacidiopycnis washingtonensis* and *Sphaeropsis pyriputrescens*.	[36]
End-point PCR	*Exobasidium maculosum*	Blueberry	USA	Morphological and phylogenetic characterization of an emerging *Exobasidium* species causing leaf and fruit spot of blueberry.	[50]
Multiplex PCR	*Fusarium* *Verticillioides* and *F. subglutinans*	Maize	Brazil	Development of gaoB-based PCR and multiplex PCR for simultaneous detection of *Fusarium verticillioides* and *F. subglutinans*.	[51]
Multiplex PCR	*Neofabraea alba*, *N. perennans* and *N. keinholzii*	Apple	Poland	Genetic diversity analysis of *Neofabraea* spp. using β-tubulin sequencing and ISSR-PCR.	[52]
qPCR	*Ramularia* *collo-cygni*	Barley	Argentina	Molecular detection of *Ramularia collo-cygni* in barley seeds and leaves using real-time PCR.	[53]
qPCR	*Pyrenophora tritici-repentis* and *Parastagonospora nodorum*	Wheat	Australia	Development of a duplex qPCR assay for specific detection and quantification of fungal species in wheat.	[54]

**Table 2:** Comparison of molecular detection assays for fungal plant pathogens.

Detection Method	Pathogen	Host	Sensitivity	Specificity	Time	Reference
LAMP, PCR, nested PCR, RT-qPCR	*Alternaria solani*	Potato; tomato	LAMP: 1.36 × 10^2^ to 1.36 ng/μL^−1^; PCR: 1.36 × 10^2^ to 1.36 × 10^−1^ ng/uL^−1^; Nested PCR: 1.36 × 10^−1^ ng/uL^−1^; RT-qPCR: 1.36 × 10^2^ to 1.36 × 10^−3^ ng mL^−1^	LAMP specificity higher than qPCR	<60 min	[64]
PCR, LAMP, qPCR, qLAMP	*Aspergillus flavus*	Peanut; dried food	PCR: 50 ng LAMP: 5 ng qPCR: 5 pg qLAMP: 5 pg	100% specificity	Rapid	[65]
qPCR; LAMP	*Ustilago tritici*	Wheat	qPCR: 10 pg/µL LAMP: 100 fg/µL	qPCR has better specificity	Rapid	[66]
qPCR-based high-resolution melting	*Sclerotium (=Agroathelia) rolfsii*; *S. delphinii*	Tomato	1 pg DNA	Highly specific. Ability to discriminate *S. rolfsii* G1, *S. rolfsii* G2, and *S. delphinii*	Rapid	[67]
Multiplex High-resolution melting Assay (Post PCR)	*Colletotrichum; Phytophthora*; *Macrophomina phaseolina*	Strawberry	1 pg DNA/10 μL (*Colletotrichum*) 1 pg DNA/10 μL (*Phytophthora*) 100 pg DNA/10 μL (*M. phaseolina*)	Highly specific.	Rapid	[68]
LAMP	*Phytophthora* sp.; *P. cactorum*	Strawberry	0.3 ng/µL to 3 pg/µL (*Phytophthora* sp.) 300 fg/µL (*P. cactorum*)	Highly specific.	Rapid	[69]

**Table 3:** Assessment of RNA-based approaches in terms of efficiency, delivery method and biosafety.

Technology	Efficiency	Delivery	Biosafety	Reference
Conventional RNAi	73% reduction of *S. sclerotiorum*	Virus-mediated (bean pod mottle virus)	Gene-specific; Lesser off-target effects	[186]
HIGS	70% and 60% reduction in petal forming lesions and sclerotia, respectively (*S. sclerotiorum*)	Transgenic plant expressing dsRNA	Minimal off-target effects on non-target organisms	[187]
SIGS	Significant reduction in disease symptoms (*B. cinerea*)	Topical spray/dipping	Rapid environmental RNA degradation limits persistence. Pathogen-specific uptake restricts non-target exposure	[188]
SIGS	30–46% reduction in powdery mildew (*Golovinomyces orontii*–*Arabidopsis thaliana* pathosystem)	Spraying dsRNA	Pathogen gene-specific RNA targeting limits non-target effects. SIGS-based suppression minimizes environmental chemical load	[189]
CRISPR/Cas9	Successful gene *VvMLO3*-edited grapevine linked to enhanced resistance against *Erysiphe necator*	*Agrobacterium tumefaciens strain* GV 3101-mediated	Reduced reliance on chemical fungicides lowers environmental risk. Requires off-target mutation assessment for biosafety assurance	[190]

**Table 4:** RNAi-based strategies for the management of fungal-driven plant disease, explained by crop, target gene, method and outcome.

Crop	Pathogen	Target Gene(s)	Application Method	Outcome	Reference
Wheat; cucumber; barley; soybean	*Fusarium asiaticum*, *F. graminearum*, *F. tricinctum*, *F. oxysporum*, *F. fujikuroi*, *Botrytis cinerea*, *Magnaporthe oryzae*, and *Colletotrichum truncatum*	β2-Tubulin	Foliar dsRNA spray	Strongly inhibits the growth of fungal pathogens	[203]
Wheat	*F. asiaticum*	Myo5	dsRNA spray (with/without fungicide)	Severe hyphal deformation and restricted mycelial growth. Synergistic antifungal effect with phenamacril	[204]
Wheat	*F. culmorum*	TRI5	SIGS and VIGS	Reduction of the proportion of infected spikelet by 73%	[205]
Wheat	*F. culmorum*	FcFgl1; FcFmk1; FcGls1; FcChsV	HIGS	Reduction (50–60%) of fusarium head blight symptom	[206]
Tomato	*F. oxysporum f.* sp. *lycopersici*	FoFLP1; FoFLP2; FoFLP3; FoFLP4; FoFLP5	RNAi (*Agrobacterium*-mediated)	Considerable reduction in disease severity	[207]
Soybean	*M. phaseolina*	MpGLS2	Exogenous siRNA	Significant reduction in mycelial growth	[208]
Strawberry	*B. cinerea*	DCL1; DCL2	Foliar dsRNA spray	At 4 dpi, BcDCL1/2 dsRNA and fungicide application showed comparable early control (≈17–20% vs. 18%), but fungicide provided superior long-term suppression by 14 dpi.	[209]
Grapevine	*B. cinerea*	BcCYP51; Bcchs1; BcEF2	High pressure spraying of leaves; petiole adsorption of dsRNAs; postharvest spraying of bunches	Reduced pre/post-harvest virulence of pathogen	[210]

## Data Availability

No new data were created or analyzed in this study. Data sharing is not applicable to this article.
